# Frequency of antimicrobial resistance among invasive and colonizing Group B Streptococcal isolates

**DOI:** 10.1186/1471-2334-6-57

**Published:** 2006-03-20

**Authors:** Stephanie M Borchardt, Joan H DeBusscher, Patricia A Tallman, Shannon D Manning, Carl F Marrs, Terrence A Kurzynski, Betsy Foxman

**Affiliations:** 1 Department of Epidemiology, School of Public Health, University of Michigan, Ann Arbor, Michigan, USA; 2 Wisconsin State Laboratory of Hygiene, University of Wisconsin, Madison, Wisconsin, USA

## Abstract

**Background:**

Group B *Streptococcus *(GBS) remains susceptible to penicillin, however, resistance to second-line antimicrobials, clindamycin and erythromycin, has increased since 1996. We describe the age-specific antibiotic susceptibility profile and capsular type distribution among invasive and colonizing GBS strains.

**Methods:**

We tested 486 invasive GBS isolates from individuals of all ages collected by a Wisconsin surveillance system between 1998 and 2002 and 167 colonizing strains collected from nonpregnant college students during 2001 in Michigan. Antimicrobial susceptibility testing was performed by disk diffusion or Etest and capsular typing was performed using DNA dot blot hybridization

**Results:**

20.0% (97/486) of invasive and 40.7% (68/167) of colonizing isolates were resistant to clindamycin (*P *< .001) and 24.5% (119/486) of invasive and 41.9% (70/167) of colonizing isolates were resistant to erythromycin (*P *< .001). Similarly, 19.8% (96/486) of invasive and 38.3% (64/167) of colonizing isolates were resistant to both antimicrobial agents (*P *< .001). 29.4% (5/17) of invasive isolates from persons 18–29 years of age and 24.3% (17/70) of invasive isolates from persons 30–49 years of age were resistant to clindamycin. Similarly, 35.3% (6/17) of invasive isolates from persons 18–29 years of age and 31.4% (22/70) of invasive isolates from persons 30–49 years of age were resistant to erythromycin. 34.7% (26/75) of invasive isolates from persons < 1 year of age were capsular type Ia, whereas capsular type V predominated among isolates from adults.

**Conclusion:**

Clindamycin and erythromycin resistance rates were high among isolates colonizing nonpregnant college students and invasive GBS isolates, particularly among the colonizing isolates. Susceptibility profiles were similar by age although the proportion of clindamycin and erythromycin resistance among invasive isolates was highest among persons 18–49 years of age. Increasing antimicrobial resistance has implications for GBS disease treatment and intrapartum prophylaxis among penicillin intolerant patients.

## Background

Group B *Streptococcus *(GBS) (*Streptococcus agalactiae*) is a significant cause of neonatal sepsis and meningitis and of severe infections in pregnant women and nonpregnant adults with underlying medical conditions [1]. GBS is also a commensal that colonizes the gastrointestinal and genitourinary tracts. The prevalence of GBS colonization among pregnant and nonpregnant adults has been estimated at 10% to 40% [2-4]. Transmission from a colonized pregnant woman to her neonate occurs via the ascending route during labor and delivery [5]. Administration of intrapartum antimicrobial prophylaxis (IAP) to colonized women has resulted in a striking decline in cases of early-onset and maternal GBS disease [6]. However, a similar decrease has not been observed for infants with late-onset disease or nonpregnant adults; currently there is no established prevention protocol for either group.

Although GBS remains sensitive to penicillin, the preferred agent for GBS infections and IAP, an estimated 12% of pregnant women report having a penicillin allergy [7], requiring the use of an alternative agent. Resistance to the second-line antibiotics clindamycin and erythromycin has increased since 1996 [7-9]. Resistance frequencies currently range from 6% to 21% for clindamycin and 12% to 29% for erythromycin in the United States [10-12], while other countries report higher rates [13].

We describe the age-specific antimicrobial susceptibility profile and capsular type distribution for invasive GBS strains from individuals of all ages, collected by the Wisconsin public health surveillance system. For comparison we included the same information for a collection of strains from colonized nonpregnant college students 18–19 years of age. Our goal was to assess whether there are differences in susceptibility profiles between colonizing and invasive GBS strains by capsular type. We hypothesized that antimicrobial susceptibility profiles and capsular types might vary with age. Continuous monitoring of GBS antimicrobial resistance patterns through surveillance activities and epidemiologic studies will help guide prophylaxis regimens for penicillin intolerant patients.

## Methods

### Invasive and colonizing strains

486 invasive strains were collected from 482 patients through the Wisconsin Division of Public Health, Bureau of Communicable Diseases, Invasive Bacterial Laboratory Surveillance System between 1998 and 2002. Forty-nine percent (234/482) of patients were female. GBS isolates were shipped by courier from hospital and regional clinical microbiology laboratories to the Wisconsin State Laboratory of Hygiene for initial processing and inclusion in the surveillance system. Invasive GBS disease was defined as any GBS infection identified from a normally sterile site. Approximately 88% of Wisconsin clinical laboratories with invasive bacterial testing capabilities voluntarily submitted isolates. Four of the 482 patients had two different GBS morphologies isolated from their culture. In each instance the two morphologies displayed different capsular types; both morphologies from each patient isolate were included in the analysis.

167 colonizing strains, previously described by our group [14], were included for comparison. Briefly, 882 colonizing strains were collected from 57 healthy male and 95 healthy, nonpregnant female college students between January and February of 2001 as part of a cross-sectional survey. After obtaining written informed consent at enrollment, a study recruiter collected throat and mouth swabs. Participants self-collected initial-void urine and anal orifice specimens, and women collected vaginal specimens using a tampon. Following enrollment, all GBS-positive participants and a random sample of those negative for GBS were invited to partake in the four follow-up visits. Participants were followed at three-week intervals for a total of 12 weeks. At each follow-up visit participants provided urine, anal orifice and vaginal (if relevant) specimens. Additional throat swabs were obtained at one of the four follow-up visits. The Health Sciences Institutional Review Board at the University of Michigan approved the study protocol (IRB file number 4133).

PFGE was performed on 882 GBS isolates cultured from the anal orifice, vagina, urine, throat, and mouth. Separate dendrograms were constructed, using BioNumerics software (Applied Maths, Kortrijk, Belgium), for each participant to determine which isolates were unique. An isolate was considered unique if it had > 3 bands that were different from the other isolates from that individual. All unique isolates from each individual were selected for further analysis (*n *= 167).

### Antimicrobial susceptibility testing

Each GBS strain was tested for susceptibility to 10 antibiotics. Disk diffusion was used to determine the susceptibilities to ampicillin (10 μg), cefazolin (30 μg), imipenem (10 μg), levofloxacin (5 μg), linezolid (30 μg), penicillin (10 IU), quinupristin-dalfopristin (15 μg), and vancomycin (30 μg) (Baltimore Biological Laboratories [BBL], Sparks, MD). GBS was subcultured onto trypticase soy agar (TSA) with 5% sheep blood (BBL, Sparks, MD) from frozen culture stocks. Disk diffusion was performed as described previously [15], while the minimum inhibitory concentration (MIC) was determined for clindamycin and erythromycin using Etest strips (AB Biodisk, NA, Piscataway, NJ). Clinical and Laboratory Standards Institute (CLSI, formerly NCCLS) guidelines were used to interpret disk diffusion and MIC results [16]. The following breakpoints were used for cefazolin (S, ≥ 28 mm; I, 26–27 mm and R, ≤ 25 mm) [7] and for imipenem (S, ≥ 30 mm).

If an isolate was erythromycin-resistant and clindamycin-susceptible, we performed a disk induction test or a "D test", by placing clindamycin (2 μg) and erythromycin (15 μg) disks 15 mm apart and observing for the induction of clindamycin resistance. The current recommendation is 12 mm [17], however these guidelines were not available when this testing was performed. An isolate with a clindamycin disk diffusion zone blunted on the side closest to erythromycin was considered inducible.

At the time of our study the CDC and CLSI recommended disk diffusion testing in 5% CO_2 _for GBS [8,16]. However, the "pH effect" of CO_2 _incubation reportedly reduces zone sizes (or increases MIC results) for macrolides and has altered susceptibility interpretations for other organisms [18,19]. To compare antibiotic activity in O_2 _to that in CO_2_, we tested 36 replicate samples of our control strain, *S. agalactiae *(ATCC 12403), and 18 clinical isolates for susceptibility to multiple antibiotics; half in ambient air and half in 5% CO_2_. Although statistically different, the mean differences in zone sizes for replicate tests in ambient air compared to 5% CO_2 _did not exceed a normal expected zone size variation of 2 mm [20]. This was also true for differences between technologists with the exception of imipenem where the mean difference was 2.33 mm. Thus the observed variation by growth condition is similar to the expected variation by technologist. In short, the use of CLSI interpretations for zone sizes listed in standard M100-S12, Table 2H, M2-Disk Diffusion, originally generated with growth in 5% CO_2_, can be used for susceptibility testing in either environment. Thus, we used ambient air for performing antimicrobial susceptibility testing of all strains including our control strain.

### Capsular typing

The capsular type was determined using DNA dot blot hybridization as described previously [21]. For a subset of isolates, however, DNA dot blot hybridization was performed with an alternative anti-fluorescein-AP antibody, according to the manufacturer's protocol (Roche Diagnostics, Penzberg, Germany); the reagents used previously were discontinued. Briefly, we used DNA dot blot hybridization with PCR-generated probes from the GBS capsular genes for serotypes Ia, Ib and II to VIII. PCR primers were designed to amplify type-specific GBS capsular gene sequences. Gene probes were constructed from the PCR products and subsequently used to classify isolates as capsular type Ia, Ib or II to VIII based on hybridization profiles. Nontypeable isolates did not bind to any of the capsular gene probes, but were probed for the presence of the GBS 16S RNA gene to verify that chromosomal DNA was present on the membrane when it was initially probed with the capsular-specific gene probes.

Three isolates displayed a gene probe homology that was not consistent with any of the nine serotypes and therefore a capsular type could not be assigned based on the DNA dot blot hybridization profile. These isolates were termed variable type (VT).

### Statistical analysis

The χ^2 ^test was used to examine the frequency of resistance between the two collections and between resistance and capsular type or age, while the χ^2 ^test for trend was used to examine the differences in the frequency of resistance between invasive strains isolated in different years. A Fisher's Exact test was used to examine associations when stratified data was sparse.

## Results

We examined the antimicrobial susceptibility profile and capsular type distribution for 486 invasive GBS isolates and 167 isolates colonizing nonpregnant adults. Most invasive strains were isolated from blood (88.4%) or CSF (3.1%). Colonizing strains were isolated from the anal orifice (59.3%), vagina (17.4%), urine (14.4%) or throat (9.0%). All invasive and colonizing isolates, with the exception of four cefazolin-intermediate isolates and three levofloxacin-resistant isolates, were universally susceptible to ampicillin, cefazolin, imipenem, levofloxacin, linezolid, penicillin, quinupristin-dalfopristin and vancomycin (Table 1). Among invasive isolates, the MIC range for clindamycin was 0.032 to > 256 μg/mL, while erythromycin was 0.023 to > 256 μg/mL. MIC ranges were similar for colonizing strains. 20.0% (97/486) of invasive and 40.7% (68/167) of colonizing isolates were resistant to clindamycin (*P *< .001) and 24.5% (119/486) of invasive and 41.9% (70/167) of colonizing isolates were resistant to erythromycin (*P *< .001). 37.1% (36/97) of invasive and 20.6% (14/68) of colonizing isolates resistant to clindamycin were identified by D-test. Furthermore, 19.8% (96/486) of invasive and 38.3% (64/167) of colonizing isolates were resistant to both clindamycin and erythromycin (*P *< .001). Slightly more clindamycin-resistant invasive isolates were also resistant to erythromycin, when compared to colonizing strains (99.0% vs. 94.1%, *P *= .09, Fisher's exact test), whereas, a significantly greater number of erythromycin-resistant colonizing strains were also resistant to clindamycin, when compared to invasive isolates (91.4% vs. 80.7%, *P *< .05).

**Table 1:** Antimicrobial susceptibility profiles for invasive (Wisconsin 1998–2002) and colonizing (Michigan 2001) group B streptococcal isolates

	**Invasive isolates (*n *= 486)**			**Colonizing isolates (*n *= 167)**		
		
**Antibiotic**	**Susceptible %**	**Intermediate %**	**Resistant %**	**Susceptible %**	**Intermediate %**	**Resistant %**
**Amplicillin**	100	0	0	100	0	0
**Cefazolin**	99.4	0.6	0	99.4	0.6	0
**Clindamycin**	80.0	0	20.0	59.3	0	40.7^1^
**Erythromycin**	75.5	0	24.5	58.1	0	41.9^1^
**Imipenem**	100	0	0	100	0	0
**Levofloxacin**	99.6	0	0.4	99.4	0	0.6
**Linezolid**	100	0	0	100	0	0
**Penicillin**	100	0	0	100	0	0
**Quinupristin-dalfopristin**	100	0	0	100	0	0
**Vancomycin**	100	0	0	100	0	0

^1^: Significantly different (*P *<.001) from the value for invasive isolates

### Resistance by year of isolation

In 2001, the year colonizing isolates were collected, 24.8% (29/117) of invasive isolates and 40.7% (68/167) of colonizing isolates were clindamycin-resistant (*P *< .01). During this same year 29.1% (34/117) of invasive isolates and 41.9% (70/167) of colonizing isolates were erythromycin-resistant (*P *< .05).

**Figure 1:**
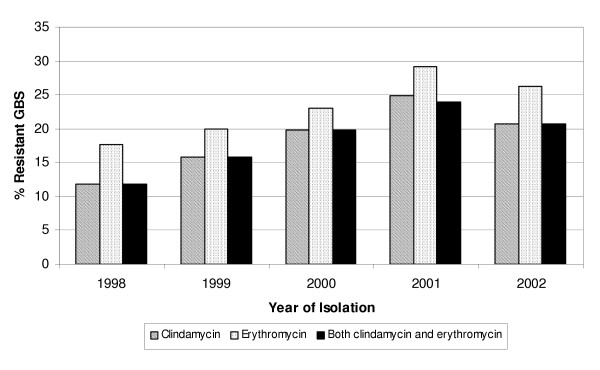
Clindamycin and erythromycin resistance among GBS invasive strains (*n *= 486) by year of isolation. Wisconsin, 1998 – 2002.

Among invasive strains, the frequency of clindamycin resistance increased from 11.8% (4/34) in 1998 to 24.8% (29/117) in 2001 and decreased slightly to 20.8% (31/149) in 2002 (*P *= .37). Similarly, the frequency of erythromycin resistance increased from 17.6% (6/34) in 1998 to 29.1% (34/117) in 2001 and decreased slightly to 26.2% (39/149) in 2002 (*P *= .47) (Figure 1). Overall, resistance frequencies among invasive isolates for each year were lower when compared to colonizing isolates. Thus, for the remainder of the analyses we compared colonizing isolates to all invasive isolates.

### Resistance by capsular type

In general, most resistant isolates were resistant to both clindamycin and erythromycin. Among invasive isolates, resistance to both antimicrobial agents ranged from 3.4% for type Ia strains to 39.4% for type V strains, and among colonizing isolates resistance to both antimicrobial agents ranged from 8.7% for type Ib strains to 63.3% for type V strains (excluding type IV strains) (Table 2). Additionally, the frequency of resistance to both antimicrobial agents differed significantly between invasive and colonizing strains for capsular types Ia, II, III and V.

39.4% (71/180) of invasive capsular type V strains and 63.3% (38/60) of colonizing type V strains were resistant to clindamycin (*P *< .01). Furthermore, 41.7% (75/180) of invasive capsular type V strains and 65.0% (39/60) of colonizing type V strains were resistant to erythromycin (*P *< .01).

### Resistance by age

The majority of invasive GBS isolates were from persons 50 years of age or older (65.4%) (Table 3). Resistance frequencies were highest among persons 18–49 years and were similar for infants with early-onset and late-onset disease (Data not shown). When compared to colonizing isolates from persons 18–19 years, resistance to clindamycin (25.3% vs. 40.7%, *P *< .05) and erythromycin (32.2% vs. 41.9%, *P *= .13) was notably lower among invasive isolates from persons 18–49 years. Furthermore, 25.3% (22/87) of invasive isolates from persons 18–49 years and 38.3% (64/167) of colonizing isolates were resistant to both antimicrobial agents (*P *< .05). Because the sample size of persons 18–29 years was inadequate to detect a significant difference between the two populations we combined the 18–29 year-old and 30–49 year-old age categories, which displayed similar frequencies of clindamycin and erythromycin resistance, allowing us to compare resistance levels for similar age groups.

**Table 2:** Clindamycin and erythromycin resistance among invasive (Wisconsin 1998–2002) and colonizing (Michigan 2001) group B streptococcal isolates by capsular type

	**Invasive isolates (*n *= 486)**			
**Capsular type**	**No. of strains**	**Clindamycin resistance only no. (%)**	**Erythromycin resistance only no. (%)**	**Resistance to both no. (%)**

**Ia**	118	0	14 (11.9)	4 (3.4)
**Ib**	54	0	0	8 (14.8)
**II**	65	0	3 (4.6)	7 (10.8)
**III**	62	1 (1.6)	2 (3.2)	5 (8.1)
**IV**	1	0	0	0
**V**	180	0	4 (2.2)	71 (39.4)
**VI**	0	0	0	0
**VII**	0	0	0	0
**VIII**	0	0	0	0
**NT**	3	0	0	1 (20.0)
**VT**	3	0	0	0

**Total**	**486**	**1 (0.2)**	**23 (4.7)**	**96 (19.8)**

	**Colonizing isolates (*n *= 167)**			

**Capsular type**	**No. of strains**	**Clindamycin resistance only no. (%)**	**Erythromycin resistance only no. (%)**	**Resistance to both no. (%)**

**Ia**	31	2 (6.5)	3 (9.7)	7 (22.6)^1^
**Ib**	23	0	1 (4.4)	2 (8.7)
**II**	18	0	0	6 (33.3)^1^
**III**	22	2 (9.1)	0	6 (27.3)^1^
**IV**	2	0	0	2 (100)
**V**	60	0	1 (1.7)	38 (63.3)^1^
**VI**	0	0	0	0
**VII**	0	0	0	0
**VIII**	1	0	0	0
**NT**	10	0	1 (10.0)	3 (30.0)
**VT**	0	0	0	0

**Total**	**167**	**4 (2.4)**	**6 (3.6)**	**64 (38.3)**

^1^: Significantly different (*P *< .05) from the value for invasive isolates.

**Table 3:** Frequency of clindamycin and erythromycin resistance among invasive and colonizing group B streptococcal isolates by age

	**Invasive isolates (*n *= 486)**		**Colonizing isolates^1 ^(*n *= 167)**	
		
**Age group (no.)**	**Clindamycin resistance no. (%)**	**Erythromycin resistance no. (%)**	**Clindamycin resistance no. (%)**	**Erythromycin resistance no. (%)**
**< 1 (75)**	16 (21.3)	18 (24.0)	-	-
**1–17 (5)**	1 (20.0)	1 (20.0)	-	-
**18–29 (17)**	5 (29.4)	6 (35.3)	68(40.7)^2^	70 (41.9)^3^
**30–49 (70)**	17 (24.3)	22 (31.4)	-	-
**≥ 50 (315)**	58 (18.4)	72 (22.9)	-	-

**Total (482)**	**97 (20.1)**	**119 (24.7)**	**68 (40.7)^2^**	**70 (41.9)^2^**

^1^: All colonizing isolates were from individuals 18–19 years of age.

^2^: Significantly different (*P *< .05) from the value for invasive isolates.

^3^: Not significantly different (*P *= .13) from the value for invasive isolates.

### Capsular type by age

Thirty-five percent (26/75) of invasive GBS isolates from persons ≤ 1 year of age were capsular type Ia, followed by type III (25.3%), and type V (20.0%) (Table 4). Similarly, 40.0% (2/5) of invasive isolates from persons 1–17 years of age were capsular type Ia, followed by one isolate each for types Ib, III, and V. Forty-one percent (164/402) of invasive isolates from persons ≥ 18 years of age were capsular type V, followed by type Ia (22.4%), and type II (14.2%). Similarly, 35.9% (60/167) of colonizing GBS isolates from persons 18–19 years of age were capsular type V, followed by type Ia (18.6%), and type Ib (13.8%).

**Table 4:** Capsular type distribution among invasive and colonizing group B streptococcal isolates by age

**Age group**	**Capsular type**										
**Invasive isolates (*n *= 486)**	**Ia** **no. (%)**	**Ib** **no. (%)**	**II** **no. (%)**	**III** **no. (%)**	**IV** **no. (%)**	**V** **no. (%)**	**VI no. (%)**	**VII no. (%)**	**VIII no. (%)**	**NT no. (%)**	**VT no. (%)**
	
**<1 (75)**	26 (34.7)	6 (8.0)	8 (10.7)	19 (25.3)	0	15 (20.0)	0	0	0	0	1 (1.3)
**1–17 (5)**	2 (40.0)	1 (20.0)	0	1 (20.0)	0	1 (20.0)	0	0	0	0	0
**18–29 (17)**	4 (23.5)	2 (11.8)	4 (23.5)	2 (11.8)	0	5 (29.4)	0	0	0	0	0
**30–49 (70)**	18 (25.7)	8 (11.4)	10 (14.3)	6 (8.6)	0	28 (40.0)	0	0	0	0	1 (1.4)
**≥ 50 (315)**	68 (21.6)	37 (11.7)	43 (13.7)	34 (10.8)	1 (0.3)	131 (41.6)	0	0	0	3 (1.0)	1 (0.3)
**Total**	118 (24.3)	54 (11.1)	65 (13.4)	62 (12.8)	1 (0.2)	180 (37.0)	0	0	0	3 (0.6)	3 (0.6)
**Colonizing isolates (*n *= 167)**											
**18–19**	31 (18.6)	23 (13.8)	18 (10.8)	22 (13.2)	2 (1.2)	60 (35.9)	0	0	1 (0.6)	10 (6.0)	0

### Resistance to other antimicrobial agents

We identified two invasive isolates and one colonizing isolate resistant to levofloxacin, which has only recently been described for GBS [22] (Data not shown). Invasive isolates were capsular types V and III, and the colonizing strain was type Ia. Invasive organisms were isolated from blood and the colonizing strain, from urine. Patients with invasive disease were 53- and 36-years of age, and their isolates were collected in 2001 and 2002, respectively. The colonized participant was 18-years of age and the year of collection was 2001.

Additionally, we identified four strains with cefazolin-intermediate resistance; all four strains were susceptible to ampicillin and penicillin. Three of these isolates were invasive isolates and one was a colonizing isolate. Each isolate was capsular type V. Invasive organisms were isolated from blood and bone cultures, and the colonizing isolate was from the vagina. Infected patients were 2 months, 47- and 70-years of age, and their isolates were collected in 1999, 2001 and 2000, respectively. The colonized participant was 19-years of age and the year of collection was 2001.

## Discussion

*In vitro *resistance to clindamycin and erythromycin has significantly increased since the implementation of IAP neonatal disease prevention strategies in 1996 [6]. Furthermore, cefazolin-intermediate resistance and levofloxacin [22] resistance among GBS has emerged. Cefazolin-intermediate resistance is concerning given that cefazolin is a first-generation cephalosporin, which shares pharmacokinetic properties with penicillin [7]. Increased and continued use of IAP, theoretically, may promote the development of GBS resistance to penicillin. Therefore IAP should be considered an interim solution to early-onset GBS disease.

We observed resistance rates of 40.7% to clindamycin and 41.9% to erythromycin among GBS isolates colonizing nonpregnant college students. Antimicrobial susceptibility profiles were quite similar by age, although levels of clindamycin (25.3%) and erythromycin (32.2%) resistance were highest among invasive isolates from persons 18–49 years of age with invasive GBS disease. This finding is of great concern.

We also found an association between clindamycin and erythromycin resistance and GBS capsular type V, which is consistent with previous reports [11,12,23-25]. Thirty-nine percent and 41.7% of invasive capsular type V strains were resistant to clindamycin and erythromycin, respectively, compared to 63.3% and 65.0% among colonizing type V strains. Capsular type V predominated among invasive isolates from persons ≥ 18 years of age and colonizing isolates from persons 18–19 years of age. It appears that capsular type V strains contribute to the high resistance rates in both populations. However, we did observe significantly higher proportions of clindamycin and erythromycin resistance among commensal than invasive isolates of serotypes Ia, II and III. Whether this represents local variation in resistance due to differences in antibiotic use, or population structure is uncertain. Nonetheless, the observation that resistance rates can exceed one-third of all strains – whether in colonizing or invasive isolates, is troubling.

Our comparison was limited in that the populations were not from the same geographic location, they had a different age distribution and isolates were collected over a similar but not identical time period. To ameliorate these inequalities we examined both populations for 2001 and compared antimicrobial resistance frequencies across similar age groups. Local variations in resistance may exist, therefore higher levels of resistance among colonizing isolates may reflect local variation. Furthermore, invasive isolates were collected through surveillance and colonizing strains as part of a cross sectional survey. Our cross sectional population was young (18–19 years), primarily white and from middle or upper income levels, potentially limiting our generalizability.

## Conclusion

Levels of clindamycin and erythromycin resistance were high among isolates colonizing nonpregnant college students and invasive GBS isolates, particularly though among the colonizing isolates. Comparisons between similar populations yielding colonizing and invasive strains would be helpful to further examine this issue. High level antimicrobial resistance among colonized, nonpregnant college students is likely driven by antibiotic use, coupled with high transmission probabilities. However, further analyses are needed to identify predictors of resistance among this population. Our current findings highlight the need for routine susceptibility testing of GBS, particularly in individuals with penicillin allergy, to ensure proper therapy.

## Competing interests

The author(s) declare that they have no competing interests.

## Authors' contributions

SB conceived of the study, participated in its design and coordination, performed capsular typing, performed the statistical analyses, and drafted the manuscript. JD performed the antimicrobial susceptibility testing and assisted with data entry. PT maintained GBS isolate collections. SM assisted with interpretation of data and critically revised the manuscript. CM participated in the design of the study and critically revised the manuscript. TK facilitated receipt of GBS isolates. BF participated in the design of the study, assisted with interpretation of the data, and critically revised the manuscript. All authors read and approved the final manuscript.

## Pre-publication history

The pre-publication history for this paper can be accessed here:


